# History of Traumatic Brain Injury Does Not Influence Rate of Progression of Clinical or Pathological Outcomes in Two Early Parkinson's Disease Cohorts

**DOI:** 10.1111/ene.70090

**Published:** 2025-03-20

**Authors:** Angus McNamara, Irina Baetu, Lyndsey Collins‐Praino

**Affiliations:** ^1^ School of Biomedicine University of Adelaide Adelaide South Australia Australia; ^2^ School of Psychology University of Adelaide Adelaide South Australia Australia

**Keywords:** neurodegeneration, Parkinson's, progression, traumatic brain injury

## Abstract

**Background:**

A history of traumatic brain injury (TBI) is associated with an increased risk of developing neurodegenerative disorders, including Parkinson's Disease (PD). However, TBI's influences on disease progression remain underassessed. This study explored whether a history of TBI influences the progression of pathological and clinical outcomes up to 5 years of follow‐up in individuals with early PD.

**Methods:**

Longitudinal data were extracted from the Parkinson's Progression Markers Initiative (PPMI) and the PostCEPT observational study. Participants in PostCEPT had complete head injury data, while PPMI participants were eligible if they completed the head injury section of the PD Risk Factor Questionnaire (*n* = 208). Principal component analysis was used to derive composite scores of cognitive ability and mood dysfunction, with motor outcomes calculated using the Movement Disorders Society Unified Parkinson's Disease Rating Scale. Progression of clinical and pathological outcomes up to 5 years and 4 years following study entry were compared, including subset analyses in PPMI examining injury severity.

**Results:**

Individuals with a history of TBI in the PPMI dataset exhibited a younger age of onset; however, a history of TBI did not affect progression rates of any assessed variables across both cohorts. Exploratory analysis determined that injury severity significantly predicted striatal dopamine transporter binding but accounted for only a small portion of outcome variance.

**Conclusion:**

While the history of TBI was associated with earlier PD onset, it did not correspond to a differential disease course. However, given differences in TBI characterisation between cohorts, additional research must be conducted to validate these findings.

## 1 Background

A lifetime history of traumatic brain injury (TBI), defined as being sustained either in childhood or adulthood, corresponds to an elevated risk of developing several different neurodegenerative disorders, including Parkinson's Disease (PD) [1]. Extensive prior research has established the link between TBI and later PD development, with a meta‐analysis of 22 studies reporting a pooled odds ratio of 1.57 [2]. Of note, across three large prospective studies, it was found that a history of TBI with loss of consciousness (LoC) greater than one hour was associated with significantly elevated risk for both Lewy body pathology and incident PD [1]. Similarly, Gardner et al. [3] reported that even a single case of mild TBI (mTBI) is associated with a 56% higher risk of developing PD. For moderate or severe injuries, this risk increases to 83%, demonstrating that injury severity may increase risk in a dose‐dependent manner [3]. Further, age at the time of injury may also be a contributing factor, with one study reporting a 44% increased risk of developing PD over a 5–7‐year period in people sustaining a TBI after the age of 55, a finding thought unlikely to be due to reverse causation [4]. Further, while sustaining a TBI in older adulthood can increase the risk of later developing PD, so too may sustaining a TBI earlier in life. In line with this, Taylor et al. [5] reported a significant effect of age at the time of first TBI with LoC on PD risk, with an odds ratio (OR) of 1.37 (95% CI: 1.01–1.86) for every 5 years earlier that the injury occurred. Notably, within that study, the median age at the time of first injury was 15 years of age, with an interquartile range of 10–18 years old, suggesting that the brain may be particularly vulnerable to the effect of TBI during the paediatric period. Taken together, said findings align with recent evidence suggesting that the relationship between clinical trajectory and age at the time of injury are not linear, with both paediatric [6] and geriatric TBI [7] corresponding to worse clinical course relative to ages in between. Overall, such findings support the critical need to account for age at the time of injury as a potential confounder for PD risk.

In addition, several studies report pathophysiological changes akin to PD following TBI, including both abnormal alpha‐synuclein (*α*‐syn) deposits and disruption of the dopaminergic system (for a review, see [8]). *α*‐syn levels are several times higher in the cerebrospinal fluid of both adult [9] and paediatric [10] patients following severe TBI, and *α*‐syn accumulation has been found to be present in swollen axons post‐injury in post‐mortem investigations of cases of severe TBI [11]. Similarly, compared to controls, a history of TBI (ranging from 6 to 366 months post‐injury) is associated with reduced dopamine transporter (DaT) binding in both the caudate (12.2%) and putamen (9.0%), two prominent regions affected for PD [12]. These findings have also been corroborated by investigations in pre‐clinical TBI models. Acosta et al. [13] reported increased ipsilateral substantia nigra (SN) *pars compacta α*‐syn accumulation, accompanied by dopaminergic neuron loss and increased inflammation, in the controlled cortical impact (CCI) model, as well as increased *α*‐syn oligomerisation and disruption of tyrosine hydroxylase, a key enzyme for dopamine synthesis, within both the SN and striatum following mild blast injury [14]. In line with this, reduced DaT levels, with concomitant increases in neuroinflammation, have been reported in the SN at 30 days following a CCI‐induced moderate TBI in mice [15], with Hutson et al. [16] reporting a 15% ipsilateral neuronal loss within this region using a lateral fluid percussion model of moderate TBI in adult rats. Such findings suggest that TBI is associated with key pathophysiological characteristics of PD and might explain why having such a history is a critical predictor of future disease development.

Despite the well‐established link between TBI and future PD development, however, the degree to which TBI influences disease progression in people with PD (PwP) has been understudied. A longitudinal study assessing PwP with and without a history (*n* = 25 per group) of TBI determined that individuals with a history of mild–moderate TBI demonstrated more profound cognitive decline compared to those with PD without TBI over a two‐year follow‐up [17]. No differences were found between groups when assessing motor and mood profiles [17]. However, this study has a noticeably small sample size, as well as only assessing a minimal follow‐up period and therefore may effectively capture heterogeneity in disease course over time [17]. Likewise, assessment of the number of head injuries in a cohort of 3848 PwP recruited as part of the Fox Insight, 1881 of whom reported a past history of TBI (54%), determined that over a 3‐year period, the cumulative effect of having five injuries or greater corresponded to more profound cognitive decline within PD [18]. Further, a higher number of injuries corresponded to higher depression scores and worse QoL, although no associations with motor severity were reported, as well as cognition being self‐reported [18], potentially introducing reporting biases. The scarcity of standardised and consistent assessment of TBI within PD, as well as expansive follow‐up, is a significant gap, given a longitudinal study across an 11‐year follow‐up period in Alzheimer's disease, another neurodegenerative disease with a past history of TBI as a recognised risk factor [19], suggested that a history of TBI may be a vital predictor of functional progression in the disease [20]. Therefore, validation in other cohorts assessing history of TBI in PwP, with similar if not larger follow‐up periods is critically needed. To address this, the current study utilised data extracted from two different cohorts, the Parkinson's Progression Markers Initiative (PPMI) [21] and the Parkinson Research Examination of CEP‐1247 trial (PRECEPT) follow‐up PostCEPT observational study (PostCEPT) [22], to determine if a prior history of TBI was associated with differences in either pathological markers or clinical presentation in PwP at baseline, and whether such a history affected disease progression up to a 5‐year follow‐up period. Further, we conducted exploratory analyses within the PPMI cohort to assess whether injury severity influenced these outcomes. We hypothesise that a prior history of TBI will correspond to an accelerated rate of progression across several clinical and pathophysiological outcomes in PD, with increasing injury severity associated with faster progression.

## 2 Method

This analysis used data from two distinct cohorts: PPMI and PostCEPT, with the PostCEPT cohort acting as the validation cohort.

### 2.1 PPMI Cohort

The cohort of interest for this analysis were recently diagnosed (diagnosis< 2 years before baseline visit) PwP (*n* = 422), with details on selection criteria detailed in Table S1. From this pool of participants, only participants from the University of California San Francisco (UCSF) site who had completed the Parkinson's Disease Risk Factor Questionnaire (PDRFQ), which provided information on past history of head injury, were eligible for this study (*n* = 208). These participants were part of the Follow‐Up of Persons with Neurologic Disease' (FOUND) project [23].

The following data were extracted from the PPMI dataset:

### 2.2 PostCEPT Cohort

The cohort of interest for this analysis included participants enrolled in both the PRECEPT and follow‐up PostCEPT study (*n* = 635), with a detailed outline of inclusion criteria detailed in Table S3 [22]. Further, an adapted version of the FOUND protocol utilised within PPMI was applied to acquire relevant TBI information, making said cohort an appropriate validation cohort for the current work. This cohort was primarily used to validate whether the rate of progression of clinical symptoms differed in those with and without a history of head injury, with extracted data outlined in Table 2.

### 2.3 TBI Classification

Based on PDRFQ responses, PPMI participants were first categorised as either PwP who had not sustained a TBI throughout their lifetime (*n* = 128; PD‐Control) or PD participants who had previously experienced a TBI (*n* = 80; PD‐TBI). For the PostCEPT study, participants who answered yes to sustaining a head injury resulting in LoC were classified as PD‐TBI (*n* = 139), while those who did not report such a history were classified as PD‐Control (*n* = 496).

An exploratory analysis was conducted within the PPMI cohort, where the PD‐TBI cohort was then further sub‐categorised according to injury severity and number of injuries sustained, a potential moderator of the effect that TBI has on long‐term outcome progression [25]. Categorisation was based on data pertaining to LoC and number of injuries sustained in the PDRFQ, resulting in the following classifications:

1. Mild TBI (*n* = 31; PD‐mTBI)—reported a single case of TBI and LoC < 60 min.
2. Repeated mTBI (*n* = 40; PD‐rmTBI)—reported > 1 case of mTBI.
3. Moderate‐to‐severe TBI (*n* = 9; PD‐M/sTBI)—reported a single case of TBI and LoC ≥ 60 min.

Three cases were excluded from this classification due to participants experiencing more than one case of M/sTBI (*n* = 2) or providing an inadequate amount of LoC information to properly classify injury severity (*n* = 1).

### 2.4 Principal Component Analysis

Principal component analysis (PCA) was utilised to develop composite scores of cognitive ability and mood dysfunction in the PPMI cohort, using corresponding assessments detailed in Table 1. PCA was also conducted to develop a cognitive ability score for the PostCEPT cohort, albeit with fewer component assessments (Table 2). Composite scores were created for baseline cognitive ability and mood dysfunction scores, as well as for changes in cognitive ability and mood dysfunction over time by conducting PCA on the slopes (estimated via linear regression) of the assessments across all time points. Assessments in each category (mood or cognition) were highly correlated, as confirmed by loading values, justifying their conversion to a single factor (Table S4).

**TABLE 1:** Data extracted from the PPMI database and their corresponding assessments.

Category	Measures
Baseline demographics	Age Sex Education (years)
Neuroimaging	**Dopamine transporter—single photon emission computed tomography (DaT‐SPECT)** Participants were injected with radionuclide ligand DaTScan. Scans were recorded at baseline and annually up to 4‐years follow‐up, excluding at 3‐year follow‐up Specific binding ratio of mean striatum (average of left and right caudate + putamen) was the measure of interest for this analysis
Biofluid pathological markers	**Cerebrospinal fluid (CSF) biomarkers (pg/mL)** Obtained via standard lumbar puncture at baseline and annually up to 5‐years follow‐up Measured using standard ELISA protocols: Unphosphorylated alpha synuclein (*α*‐syn) Measured using Elecsys electrochemiluminescence immunoassays: Phosphorylated tau (phosphorylation site: threonine 181) (P‐Tau) β‐amyloid^1–42^ (Aβ)
Motor symptoms	**Movement Disorder Society‐Unified Parkinson's Rating Scale (MDS‐UPDRS)** A comprehensive questionnaire containing 50 items pertaining to motor and non‐motor symptoms of Parkinson's disease; combination of clinical assessment and self‐report [24]. This was extracted at baseline and at annual follow‐ups up to 5‐years while participants were on medication. The following parts were used: *Part 3: Motor examination* Rigidity score (sum of MDS‐UPDRS subitems of item 3.3) Tremor score (sum of MDS‐UPDRS subitems of items 3.15, 3.16, 3.17 & 3.18)
Cognition	Various aspects of cognition were assessed at baseline and at annual follow‐up up to 5 years. **Hopkins Verbal Learning Test (HVLT)** Verbal recognition and recall (learning and memory) **Sematic fluency (SFT)** Executive function and semantic memory **Letter number sequencing (LNS)** Working memory **Benton judgement of line orientation (JLO)** Visuospatial ability **Symbol digit modalities (SDM)** Psychomotor/processing speed
Mood	Various aspects of mood were assessed at baseline and at annual follow‐up up to 5 years. **State–trait anxiety inventory (STAI)** Anxiety (the total STAI scores were analysed) **Geriatric Depression Score (GDS)** Depression
Parkinson's Disease Risk Factor Questionnaire (PDRFQ)	The PDRFQ is used to evaluate risk factors of PD by compiling information on lifetime experiences pertaining to health and lifestyle. Of particular interest for this study was the head injury portion of this survey Questions that comprise this questionnaire are reported in Table S2

**TABLE 2:** Data extracted from the PostCEPT database and their corresponding assessments.

Category	Measures
Baseline demographics	Age Sex Education (coded as highest level of education ranges 1 = < 9th grade and 7 = Graduate or professional degree)
Motor symptoms	**MDS‐UPDRS** This was extracted at baseline and at annual follow‐ups up to 4‐years while participants were on medication. The following parts were used: *Part 3: Motor examination* Rigidity score (sum of MDS‐UPDRS subitems of item 3.3) Tremor score (sum of MDS‐UPDRS subitems of items 3.15, 3.16 & 3.17)
Cognition	Various aspects of cognition were assessed at year‐1 and at annual follow‐up up to 4 years. **Hopkins Verbal Learning Test (HVLT)** Verbal recognition and recall (learning and memory) **Sematic fluency (SFT)** Executive function and semantic memory **Letter number sequencing (LNS)** Working memory
Depression	The Geriatric Depression Scale (GDS) was assessed at baseline and at annual follow‐up, up to 4‐years
History of TBI	An adapted version of the FOUND protocol was used within the PostCEPT study, whereby participants were asked if they had sustained a TBI resulting in LoC in their lifetime. Information on injury severity or other injury characteristics were not collected

### 2.5 Statistical Analyses

All data were analysed using R (version 1.4.1717) [26]. Given distinctions in the way TBI history was ascertained in the PPMI and PostCEPT cohorts, as well as differences in the way clinical variables were measured, the datasets were not pooled together and instead treated as separate cohorts in subsequent analyses. A Shapiro–Wilk test was conducted and determined that the distribution of several variables deviated significantly from normality, with only age and baseline cognitive ability in the PPMI cohort being normally distributed. Therefore, normally distributed variables are displayed as mean ± standard deviation, whereas non‐normally distributed variables are reported as median (interquartile range). In addition, frequency data were summarised as counts (percentage). For group level comparisons, independent *T*‐tests and Mann–Whitney *U* tests were conducted when comparing history of TBI; however, when exploring injury severity, one‐way ANOVA's and Kruskal–Wallis tests were conducted, and if significance was detected, post hoc Tukey or Dunn tests were conducted for normal and non‐normally distributed data respectively, with a Bonferroni multiple comparison correction applied.

When assessing rates of progression over time, given significant deviations from normality when accounting for follow‐up timepoints, a non‐parametric equivalent of a mixed ANOVA was conducted via the *nparLD* package [27], with history of TBI or TBI severity as a between‐subjects factor and follow‐up period as a within‐subjects factor. If significant interaction effects were present, slopes were calculated using all timepoints and compared between groups via either Mann–Whitney *U* tests or Dunn tests if more than two groups were present.

Finally, simple linear regression models were conducted to assess both the history of TBI and injury severity as predictors of progression in clinical outcomes including cognitive, mood, and motor outcomes. Further, if either a significant interaction effect or a significant main effect of the history of TBI or injury severity were present in the mixed ANOVA for a specific pathological biomarker assessed, an additional linear regression model was created to assess predictors of the progression of that marker. For these models, injury severity was re‐converted into an ordinal format, ranked from least severe to most severe (1 = PD without TBI (PD‐Control); 2 = PD‐mTBI and 3 = PD‐M/sTBI), with rmTBI and mTBI being collapsed into a single group. Further, the PPMI dataset has information for individuals sustaining multiple injuries; however, the number of injuries accounted for was capped at five. It was unclear whether this was reflective of the maximum number of injuries reported within the dataset or a constraint of data collection, and therefore, the number of injuries was likewise treated as an ordinal variable (1 = no injuries; 2 = one injury and 3 = multiple injuries). An interaction term between injury severity and the number of injuries sustained was included as a predictor. Age, sex and education were controlled for in the models. All models were displayed via the *sjPlot* package [28]. The significance level for all analyses was set at *p* < 0.05.

## 3 Results

### 3.1 History of TBI

Of the 208 participants extracted from the PPMI cohort, approximately 40% (*n* = 80) reported a confirmed TBI in their lifetime. The prevalence of TBI within the PostCEPT cohort was markedly lower, at approximately 22% (*n* = 139). The duration from the time of injury (or first injury, in the case of the rmTBI group) to assessment within the PPMI cohort was 47.30 (34–54.65) years; however, minimal information was available for the time of most recent injury. Of the participants who provided information on the time since injury (*n* = 68), the majority reported sustaining a paediatric TBI, defined as a head injury throughout childhood or adolescence [29] (age at time of injury ≤ 18 years, *n* = 49), with the remainder sustaining their TBI during adulthood (age at time of injury > 18 years, *n* = 19). Demographic information for paediatric and adult TBI groups is presented in Table S5. Information on the duration from the time of injury to assessment was not available in the PostCEPT dataset.

#### 3.1.1 Baseline Comparisons

To determine whether having a history of TBI corresponded to differences in variables of interest at the time of diagnosis, statistical comparisons of demographics, striatal DaT binding, biomarker concentrations and clinical outcomes were conducted on baseline values. PD‐TBI participants were not statistically different from PD‐Control in any of our comparisons, except for age in the PPMI cohort (Table 3). Overall, PD‐TBI were significantly, *t*(158) = 2.04, *p* = 0.04 younger (59.85 ± 10.31) compared to PD‐Control (62.80 ± 9.69). Despite this, the percentage of individuals with Young‐Onset PD did not differ (*p* > 0.05) in those with or without a history of TBI in either the PPMI or PostCEPT cohort (Table 3). Further, within the PostCEPT cohort, the only significant difference was found in GDS assessment, with PD‐TBI displaying significantly higher scores (2 (1–4); *U* = 30,264, *p* = 0.04) compared to PD‐Control (2 (0.5–3)).

**TABLE 3:** Differences in variables of interest between PD‐Controls and PD‐TBI were assessed at baseline.

	PPMI cohort				*p*	PostCEPT cohort				*p*
	PD without TBI		PD with TBI			PD without TBI		PD with TBI		
	*n*	Mean ± SD/median (IQR)	*n*	Mean ± SD/Median (IQR)		*n*	Mean ± SD/median (IQR)	*n*	Mean ± SD/median (IQR)	
Age	127	62.80 ± 9.69	80	59.85 ± 10.31	**0.04**	492	63.64 ± 9.63	138	63.26 ± 9.85	0.69
Young‐onset (< 50)	15 (12%)		16 (20%)		0.55	33 (6%)		11 (7%)		0.75
Older‐onset (≥ 50)	112 (88%)		64 (80%)			459 (94%)		127 (93%)		
Education^a^	124	16 (16–18)	75	16 (15–18)	0.39	496	6 (4–7)	139	6 (4–7)	0.57
Sex										
Male	75 (59%)		53 (60%)		0.92	315 (64%)		98 (70%)		0.14
Female	53 (41%)		35 (40%)			179 (36%)		40 (30%)		
Rigidity score	128	3 (2–6)	80	3 (2–6)	0.19	496	4 (2–7)	139	4 (2–7)	0.70
Tremor score^b^	128	4 (2–7)	80	4 (2–6)	0.96	496	3 (1–4)	139	3 (1–5)	0.20
Cognitive ability^c^	123	0.08 ± 0.91	75	−0.17 ± 1.13	0.12	371	0.07 (−0.56–0.73)	107	0.01 (−0.52–0.58)	0.57
Mood dysfunction^d^	124	−0.29 (−0.73 to 0.39)	75	−0.13 (−0.54 to 0.54)	0.10	495	2 (0.5–3)	138	2 (1–4)	**0.04**
Striatal DaT binding	122	1.34 (1.13–1.61)	77	1.32 (1.02–1.52)	0.17					
*CSF α*‐syn	86	1356.1 (1034.6–1760.1)	55	1390.7 (1097.7–1711.9)	0.68					
CSF P‐Tau	110	13.51 (10.93–17.26)	72	13.57 (11.15–15.55)	0.71					
CSF Aβ	114	876.96 (684.63–1217.25)	72	863.75 (678.63–1204.23)	0.85					

*Note:* Normally distributed variables are displayed as mean ± SD, with statistical differences determined via *t*‐tests. Non‐parametric variables are displayed as median (IQR), with statistical differences determined via Mann–Whitney *U* tests. Statistically significant differences are denoted by bolded *p* values.

^a^ Education was graded differently between cohorts: PPMI = Years; PostCEPT = Highest level of education (1 = < 9th grade and 7 = Graduate or professional degree) (Tables 1 and 2).

^b^ PostCEPT did not have available data on tremor constancy; therefore, this was not included in the PostCEPT tremor score (Tables 1 and 2).

^c^ Assessments used to derive cognitive ability composites differed between cohorts (Tables 1 and 2).

^d^ PostCEPT cohort did not have available data on mood measures outside of GDS, therefore GDS alone acted as the mood dysfunction measure in that cohort.

#### 3.1.2 Rate of Progression

**FIGURE 1:**
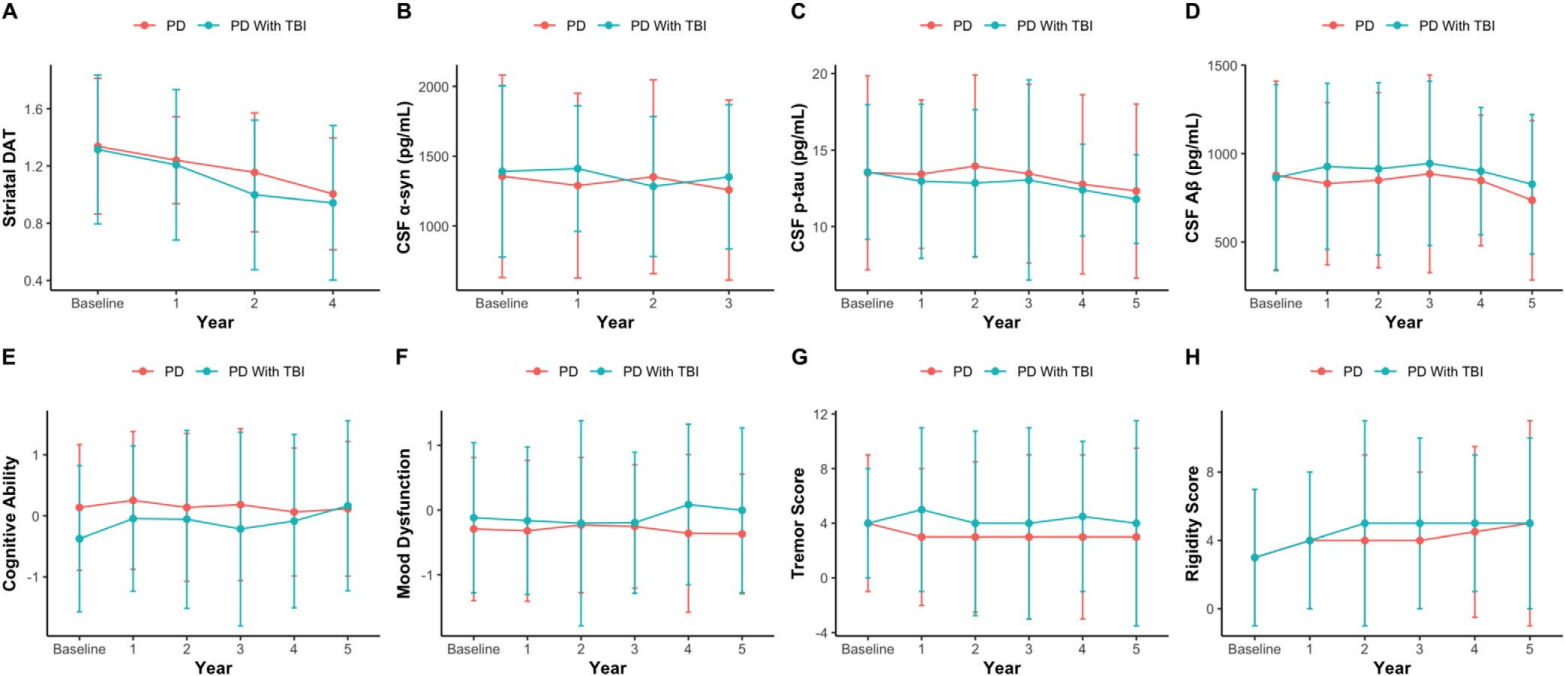
Median and IQR values are displayed at each timepoint for extracted variables of interest. No significant interaction effect was present when comparing the rate of progression in PD‐Controls compared to PD‐TBI within the PPMI cohort.

**FIGURE 2:**
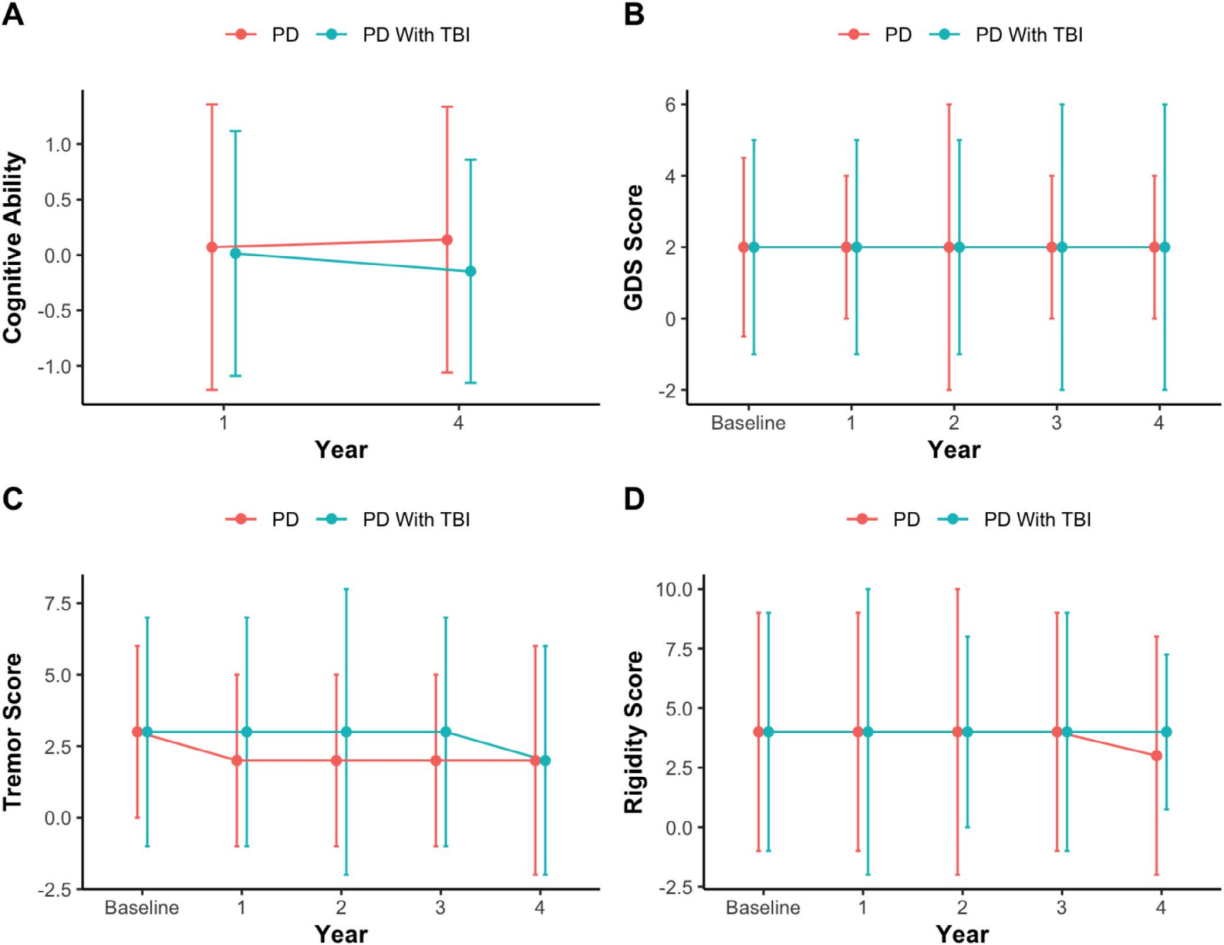
Median and IQR values are displayed at each timepoint for extracted variables of interest. No significant interaction effect was present when comparing rate of progression in PD‐Controls compared to PD‐TBI within the PostCEPT cohort.

Overall, no significant interaction effects were present in PwP with (PD‐TBI) versus without a history of TBI (PD‐Control) in either the PPMI (Figure 1) or PostCEPT cohorts (Figure 2).

#### 3.1.3 Linear Regression

Across all models, a history of TBI was not a significant predictor of any neuropsychiatric or motor outcome at follow‐up in either the PPMI (Table 4A) or PostCEPT cohorts (Table 4B). In the PPMI models including a history of TBI as a predictor, sex was a significant predictor of year‐5 cognitive ability (*β* = −0.51, *p* = 0.007) and education was a significant predictor of year‐5 tremor score (*β* = −0.18, *p* = 0.034), with no other predictor reaching statistical significance. Similarly, within the PostCEPT cohort, male sex and age were significant predictors of both year‐4 cognitive ability (sex: *β* = −0.34, *p* = 0.026; age: *β* = −0.04, *p* < 0.001) and rigidity score (sex: *β* = 1.32, *p* = 0.018; age: *β* = 0.08, *p* = 0.003), with educational level attained also acting as a significant predictor of cognitive ability (*β* = 0.18, *p* < 0.001). It is important to note, however, that the included predictors explained only a small amount of the variance across all models (range = 0.008–0.265).

**TABLE 4:** Linear regression was conducted to determine whether a history of TBI in the (A) PPMI or (B) PostCEPT cohort was a significant predictor of neuropsychiatric outcomes or motor outcomes at follow‐up.

(A) PPMI cohort												
	Year 5 cognitive ability		Year 5 mood dysfunction		Year 5 rigidity score		Year 5 tremor score		Year 3 CSF α‐syn		Year 4 striatal DaT	
Predictors	Estimates	*p*	Estimates	*p*	Estimates	*p*	Estimates	*p*	Estimates	*p*	Estimates	*p*
Intercept	0.25	0.693	−0.06	0.165	−0.06	0.077	−0.03	**< 0.001**.	−0.09	**< 0.001**	0.15	**< 0.001**
Age	−0.08	0.421	−0.09	0.280	0.04	0.609	−0.10	0.218	−0.05	0.662	0.01	0.887
Male sex	−0.51	**0.007**	−0.08	0.625	0.02	0.911	0.06	0.709	0.19	0.425	−0.13	0.480
Education	0.17	0.070	−0.08	0.353	−0.04	0.612	−0.18	**0.034**	−0.13	0.260	−0.01	0.873
History of TBI	0.10	0.591	0.26	0.124	0.12	0.504	−0.01	0.944	−0.07	0.779	−0.18	0.303
Observations	114		156		143		147		84		143	
*R* ^2^/*R* ^2^ adjusted	0.098/0.065		0.038/0.012		0.008/−0.021		0.043/0.017		0.026/−0.023		0.013/−0.016	

(B) PostCEPT cohort								
	Year 4 cognitive ability		Year 4 GDS		Year 4 rigidity score		Year 4 tremor score	
Predictors	Estimates	*p*	Estimates	*p*	Estimates	*p*	Estimates	*p*
Intercept	1.12	0.053	2.75	0.120	−1.01	0.621	1.86	0.337
Age	−0.04	**< 0.001**	0.01	0.605	0.08	**0.003**	0.01	0.588
Male sex	−0.34	**0.026**	0.25	0.600	1.32	**0.018**	0.41	0.436
Education	0.18	**< 0.001**	−0.17	0.203	0.07	0.658	0.02	0.873
History of TBI	−0.14	0.419	0.45	0.379	0.43	0.469	0.44	0.435
Observations	154		162		162		162	
*R* ^2^/*R* ^2^ adjusted	0.265/0.246		0.021/−0.004		0.110/0.087		0.013/−0.012	

*Note:* In addition, striatal DaT binding and CSF α‐syn were assessed as outcomes in the PPMI cohort at year‐4 and year‐3, respectively. All models controlled for age, education years and sex of the participants. Statistically significant differences (*p* < 0.05) are denoted by bolded *p*‐values.

Further, as GDS significantly differed between individuals with and without a history of TBI at baseline within the PostCEPT cohort, an exploratory model was conducted with baseline GDS included as a predictor (Table S6). In brief, baseline GDS significantly predicted GDS at year‐4 follow‐up (*β* = 0.73, *p* < 0.001); however, it did not significantly predict any other variable of interest.

### 3.2 Injury Severity

When accounting for injury severity within the PPMI cohort, PD‐TBI subjects were predominantly classified as rmTBI (50%; *n* = 40), followed by mTBI (37.5%; *n* = 31) and M/sTBI (12.5%; *n* = 9).

#### 3.2.1 Baseline Comparisons

While it was expected that accounting for injury severity would be associated with more profound differences between groups, there were no statistically significant differences between TBI severity groups, likely due to the small number of participants across groups, particularly for m/STBI (Table 5).

**TABLE 5:** Differences in variables of interest between PD‐controls and different severities of PD‐TBI (PD‐mTBI, PD‐rmTBI, PD‐M/sTBI) were assessed at baseline in the PPMI cohort.

	PD without TBI		PD mTBI		PD rmTBI		PD M/sTBI		*p*
	*n*	Mean ± SD/median (IQR)	*n*	Mean ± SD/median (IQR)	*n*	Mean ± SD/median (IQR)	*n*	Mean ± SD/median (IQR)	
Age	128	62.8 ± 9.69	31	61.11 ± 10.42	40	59.42 ± 10.03	9	57.38 ± 11.75	0.15
Young‐onset (< 50)	15 (12%)		4 (13%)		6 (15%)		2 (22%)		0.54
Older‐onset (≥ 50)	112 (88%)		27 (87%)		33 (85%)		7 (78%)		
Education	124	16 (16–18)	29	16 (14–17)	37	16 (16–18)	9	18 (15–19)	0.35
Sex									
Male	75 (59%)		20 (65%)		23 (57.5%)		6 (66.6%)		0.9
Female	53 (41%)		11 (35%)		17 (42.5%)		3 (33.3%)		
Striatal DaT binding	122	1.34 (1.13–1.61)	30	1.34 (1.11–1.56)	39	1.33 (1.11–1.62)	8	1.34 (1.11–1.59)	0.33
*CSF α*‐syn	86	1356.1 (1034.60–1760.08)	24	1362.7 (1040.9–1716.5)	26	1380.2 (1036.7–1780.9)	5	1382.15 (1095.95–1757.03)	0.40
CSF P‐Tau	110	13.51 (10.93–17.26)	29	13.52 (11.0–16.60)	35	13.62 (11–17.21)	8	13.43 (10.97–16.58)	0.68
CSF Aβ	114	876.96 (684.63–1217.25)	29	873.6 (661.3–1202)	36	878.35 (702.45–1224.25)	7	879.5 (682.8–1215.5)	0.35
Rigidity score	128	3 (2–6)	31	4 (3–6)	40	3 (1–6)	9	2 (2–3)	0.24
Tremor score	128	4 (2–7)	31	4 (2–6)	40	4 (2–6)	9	5 (2–7)	0.83
Cognitive ability	123	0.08 ± 0.91	29	−0.22 ± 1.29	9	−0.05 ± 0.64	37	−0.15 ± 1.11	0.40
Mood dysfunction	124	−0.29 (−0.73 to 0.39)	29	−0.32 (−0.76 to 0.18)	9	−0.07 (−0.41 to 0.65)	37	−0.19 (−0.45 to 0.15)	0.19

*Note:* Normally distributed variables are displayed as mean ± SD, with statistical differences determined via one‐way ANOVAs. Non‐parametric variables are displayed as median (IQR), with statistical differences determined via Kruskal–Wallis tests. Statistically significant differences are denoted by bolded *p* values.

#### 3.2.2 Rate of Progression

**FIGURE 3:**
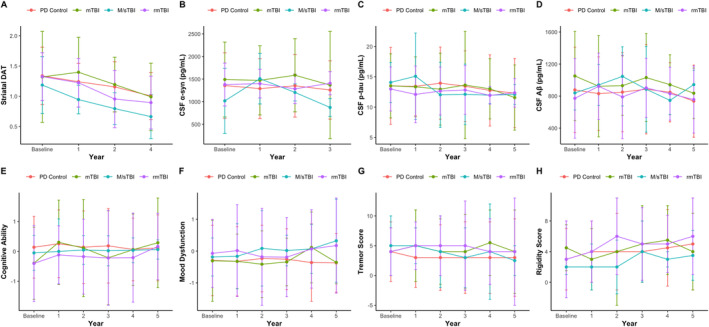
Median and IQR values are displayed at each timepoint for extracted variables of interest. While there was a significant interaction between timepoint and TBI severity for CSF α‐syn (pg/mL) (B), there was no significant difference in progression as determined via Dunn tests conducted on slopes between injury severity groups. Significant main effects for injury severity and follow‐up timepoint were present for striatal DAT binding (A); however, no other significant main or interaction effect was present for any other variable of interest.

In the TBI severity analyses, there was only a significant interaction between TBI severity and follow‐up time for CSF *α*‐syn, *F*(2, 89) = 3.25, *p* = 0.02; Figure 3B. However, a post hoc analysis of the slopes found no significant differences between severity groups (*p* > 0.05). Of note, while for striatal DaT binding there was no significant interaction between TBI severity and follow‐up period, *F*(6, 186) = 1.56, *p* = 0.20 (Figure 3A), there was a significant main effect of TBI severity, *F*(3, 87) = 3.8, *p* = 0.02, as well as follow‐up period, *F*(2, 186) = 28.6, *p* < 0.0001. Post hoc Dunn tests revealed that a negative relationship between injury severity and striatal DaT binding was present (Figure S1A), with PD‐M/sTBI (0.90 (0.74–1.14)) having significantly lower binding compared to PD‐Controls (1.21 (0.99–1.42); *Z*(4, 405) = −3.56, *p* = 0.002) and PD‐mTBI (1.20 (0.96–1.54); *Z*(4, 123) = −3.29, *p* = 0.005). Additionally, PD‐rmTBI (1.06 (0.87–1.33)) also demonstrated significantly lower binding compared to PD‐Controls, *Z*(4, 494) = −3.06, *p* = 0.009. Further, striatal DaT binding progressively deteriorated at each follow‐up timepoint (Figure S1B).

#### 3.2.3 Linear Regression

When considering injury severity (Table 6), the interaction between TBI severity and number of severities significantly predicted striatal DaT binding (*β* = −0.44, *p* = 0.047). Other significant predictors in the models included sex as a significant predictor of year‐5 cognitive ability (*β* = −0.47, *p* = 0.008) and education as a significant predictor of year‐5 tremor score (*β* = −0.18, *p* = 0.030). Similar to the models with history of TBI as a predictor, the proportion of the variance explained by the included predictors, however, was very low (*R* ^2^ range = 0.026–0.098).

**TABLE 6:** Linear regression was conducted in the PPMI cohort to determine whether injury severity was a predictor of neuropsychiatric or motor outcomes at year‐5 follow‐up, as well as striatal DaT binding and CSF α‐syn at year‐4 and year‐3, respectively.

PPMI: injury severity												
	Year 5 cognitive ability		Year 5 mood dysfunction		Year 5 rigidity score		Year 5 tremor score		Year 3 CSF α‐syn		Year 4 striatal DaT	
Predictors	Estimates	*p*	Estimates	*p*	Estimates	*p*	Estimates	*p*	Estimates	*p*	Estimates	*p*
Intercept	0.29	0.701	0.05	0.197	−0.02	0.07	−0.04	**< 0.001**	−0.11	**< 0.001**	0.07	**< 0.001**
Age	−0.07	0.430	−0.08	0.330	0.04	0.645	−0.11	0.188	−0.08	0.497	−0.01	0.964
Male sex	−0.51	**0.008**	−0.09	0.589	0.03	0.855	0.08	0.652	0.18	0.446	−0.11	0.532
Education	0.17	0.073	−0.08	0.353	−0.05	0.594	−0.18	**0.039**	−0.11	0.309	−0.01	0.892
TBI severity	0.01	0.914	0.10	0.345	−0.14	0.230	−0.16	0.172	−0.22	0.171	−0.11	0.342
Number of injuries	0.03	0.792	0.07	0.549	0.20	0.09	0.14	0.235	0.13	0.412	−0.06	0.629
TBI severity × number of injuries	−0.14	0.824	0.54	0.353	0.73	0.736	0.07	0.978	−492.23	0.318	−0.44	**0.041**
Observations	114		156		143		147		84		143	
*R* ^2^/*R* ^2^ adjusted	0.098/0.065		0.049/0.024		0.007/−0.021		0.044/0.017		0.034/−0.015		0.036/0.008	

*Note:* All models controlled for age, education years and sex of the participants. Statistically significant differences are denoted by bolded *p*‐values.

## 4 Discussion

This study was among the first to look beyond whether TBI increases the risk for PD development, and instead consider how either a history of TBI or the severity of injury may influence the progression of motor and non‐motor symptoms, as well as pathological markers, in established disease. Overall, across both the PPMI and PostCEPT early PD cohorts, aside from PD‐TBI demonstrating a significantly younger age of onset compared to PD‐Controls in the PPMI dataset, no significant differences were found in any other domain either at baseline or for the rate of symptom progression over a 5‐year follow‐up period for any variable of interest. Similarly, a history of TBI was not a significant predictor of motor, cognitive, or mood function at follow‐up timepoints in either cohort. Although the severity of injury was a significant predictor of striatal DaT binding at year‐4 follow‐up within the PPMI cohort, it accounted for only a very small proportion of the total variance. Thus, while this could have potential important implications for the clinical management of PwP with a prior history of TBI, suggesting that those with a history of more severe injury may be particularly vulnerable to greater degradation in the dopaminergic system and that there may be utility for regular monitoring of those with a history of TBI for the development of the prodromal symptoms of PD for early identification of risk, this finding should be interpreted with caution, and future research is warranted.

To date, the previous literature exploring whether a history of TBI affects disease progression in PD is limited and not entirely consistent. Crane et al. [1] reported that a history of TBI with LoC greater than one hour was associated with faster progression of Parkinsonian symptoms in two prospective cohorts (the Religious Orders Study and Memory and Ageing Project). This previous study, however, did not consider both motor and non‐motor domains, or treat these symptoms as continuous variables, as we have done here. Instead, they derived a summary global Parkinsonian sign score based on performance on a 26‐item assessment from a modified version of the motor section of the UPDRS, conducted by a trained nurse, which was re‐coded into an ordinal format as a measure of progression [1]. Thus, it may be that the operationalisation of the rate of progression outcome variable influenced the differences observed between groups. For example, the sign score incorporated other aspects of motor performance not assessed in the current work, such as bradykinesia and gait disturbance. Likewise, heterogeneity in assessments utilised may also drive differences reported between present findings and those within the Fox Insight cohort, whereby the latter reported a cumulative effect of head injury resulting in accelerated rates of cognitive decline, particularly when five or greater injuries were reported [18]. However, unlike the present study, cognition in the Fox insight study was assessed using a self‐reported scale, namely the Penn Parkinson's Daily Activities Questionnaire [30], which greatly differs from composite cognitive ability scores derived from PPMI and PostCEPT. Additionally, assessment of the Fox Insight cohort determined that greater LoC from the most severe injury did not correspond to more rapid progression of cognitive complaints [18], contrasting previous findings that suggest otherwise [1]. Taken together, inconsistencies in findings across studies may stem from a lack of standardisation in the way progression and outcomes are considered, demanding harmonisation across studies for improved translatability of research findings.

Another consideration is that Fox insight and the multi‐cohort study by Crane and colleagues included a substantially larger sample size (*n* = 3484 and 7130, respectively) compared to the present study (*n* = 208 in the PPMI cohort; *n* = 635 in the PostCEPT cohort). Therefore, it is possible that said studies possessed greater statistical power to detect differences in rates of progression between groups. Conversely, in a much smaller cross‐sectional cohort study of 69 PwP, a history of TBI was not associated with worse motor scores on the UPDRS part three [31]. However, within that study, the number of TBIs sustained was associated with poorer performance across several measures of cognition, including global cognition, executive function, memory, and language, as well as increased depressive symptoms, compared to those without such a history, an effect not seen in the current work [31]. It is important to note, however, that this study did not consider progression over time in the PD cohort, as we have done here. Additionally, the sample of PD participants in that study was more heterogeneous, being a community‐dwelling sample with larger variability in time since diagnosis (6.52 ± 4.69), while the present study consisted only of individuals within two to five years of PD diagnosis (for PPMI and the PostCEPT cohort, respectively). Given that cognitive decline tends to worsen over time in PD [32], it may be that our relatively limited follow‐up period was too short to accurately assess the effect of TBI on progression of cognitive change.

However, it should be noted that divergence within early PD populations has been reported within such timeframes [33, 34, 35], meaning rather than an insufficient follow‐up period, lack of differences may be due to the nature of the PD‐TBI cohort available for analysis in the current study. While currently the gold standard PD database, PPMI, was not designed at its outset to assess the effect of TBI on disease progression, with head injury questions instead included as part of one questionnaire given at a single site. This reduced the pool of participants available for analysis for the current work, with inconsistent collection and reporting of variables, further contributing to the relatively small sample size. This also limited the power of the subgroup analyses investigating injury severity and made it impossible to assess the effect of other variables of interest, such as the number of injuries sustained or age at the time of injury. Further, substantial differences between the rates of TBI were present between cohorts explored in the current work, with PPMI reporting a 40% incidence rate, whereas PostCEPT incidence was noticeably lower, at approximately 20%. This is likely due to the differences in head injury characterisation between cohorts. PostCEPT classified TBI as any head injury resulting in LoC; however, only approximately 10% of concussions result in LoC [36, 37]. Therefore, it is likely that the true incidence of TBI reported within the PostCEPT dataset is underrepresented. Further, such categorisation did not allow for exploration of additional variables of interest related to the injury itself, such as injury severity, an important factor to consider, as illustrated by our subgroup analyses in the PPMI cohort in the current study. To address these limitations, a more purposeful, prospective study design should be employed, specifically targeted at recruiting PwP with and without a history of head injury, with consistent characterisation of head injury and collection of additional variables of interest related to the injury.

Interestingly, when evaluating the progression of disease‐related pathology over a five‐year follow‐up in the PPMI cohort, a significant interaction between injury severity and follow‐up time was reported for CSF levels of α‐syn, although post hoc analysis did not reveal any significant differences in slopes between injury severities. This may be due to a lack of sufficient follow‐up time, with CSF levels of α‐syn only measured up to 3 years of follow‐up. It may be that, because the PPMI includes only those with newly diagnosed PD (i.e., within two years of diagnosis), this timeframe was not sufficient to fully evaluate the potential effect of TBI on pathological α‐syn progression, with longer follow‐up times needed to see more marked differences between groups. This is supported by Førland et al. [38], who reported that, over a four‐year period, no substantial change in CSF α‐syn was present in a PD cohort, although it should be noted that the sample was small. Conversely, Spiegel et al. [39] have suggested that, while TBI promotes earlier clinical onset of PD, it does so in the absence of more advanced pathology. Rather, it lowers the threshold required for pathology to manifest itself as visible symptoms. In line with this, while those with a history of TBI in the PPMI cohort demonstrated a significantly younger age of onset of disease than those without such a history (an effect that was not seen in the PostCEPT cohort), there were no significant baseline differences between groups for any of the pathological markers assessed. Despite demonstrating a significantly younger age of onset, a history of TBI did not result in increased rates of young‐onset PD relative to individuals without a history of TBI in either cohort, nor was this influenced by injury severity/number of injuries within the PPMI cohort. This is an important consideration, given that young‐onset PD displays both clinical and neuropathological distinctions to older‐onset [40], with present findings suggesting that while TBI lowers pathological thresholds required for clinical onset, it does not correspond to an increased likelihood of developing young‐onset PD.

Of note, however, is that the interaction between injury severity and the number of TBI's sustained acted as a significant predictor of greater reductions in striatal at four‐year follow‐up, despite there being no significant difference in rate of progression between groups. Additionally, there was a main effect of TBI severity reported in the mixed ANOVA, with more severe injury associated with lower striatal DaT binding. This is in line with previous literature in TBI cohorts, which has shown that TBI severity may be an important contributor to the reductions in dopaminergic levels observed on SPECT DaT imaging in TBI survivors. For example, using the I‐β‐CIT tracer, Donnemiller et al. [41] reported greater than 50% reduction in striatal DaT binding at an average of 141 days following head trauma leaving participants in a vegetative state. In studies assessing less extreme cohorts, but still M/sTBI, reductions have also been noted in striatal DaT binding, albeit to a smaller degree, ranging from 15% to 20% [42, 43]. Similarly, a higher number of TBI's has been shown to lead to more profound dopaminergic system alterations within preclinical models. A rodent model of TBI, comprised of rats aged six to seven weeks with injury induced via closed‐head momentum exchange, reported that while a single case of TBI resulted in focal changes to the caudate and putamen, rmTBI rats (three head injuries) showed widespread diffusivity alterations across white matter tracts, basal ganglia and brainstem, with the midbrain dopamine system demonstrating marked hypoconnectivity [44].

To date, however, there is very limited literature assessing concussion‐induced changes in striatal DaT binding and other measures of dopaminergic functioning in clinical populations, making it difficult to fully assess the effect of TBI severity, as well as its interaction with the number of injuries, on dopaminergic integrity. This is a considerable oversight considering 90% of TBIs are considered mild, largely reflected in the incidence of mTBI and rmTBI within the PPMI cohort, and such injuries still represent a considerable public health concern and therefore should be better represented across the literature [45]. Taken together, the present findings suggest that assessing TBI severity, and the potential confounding effects of the number of injuries, may be an important consideration for future research aiming to characterise disease progression in PwP.

It is important to note, however, that the regression models exploring the predictive value of TBI‐related factors yielded a markedly low proportion of variance explained in all outcome variables, and a history of TBI was not a significant predictor in any model. This suggests that TBI in isolation is not the sole driver of PD development and progression and that it must be considered in the wider context of other known risk factors for PD to become more clinically relevant. For example, in addition to a history of TBI, PD is also known to be associated with multiple other genetic and environmental variables, and PD pathogenesis is thought to involve complex interactions between these factors (for a review, see [46]). One such factor is pesticide exposure, with a meta‐analysis reporting that exposure substantially increases the risk of PD development [47]. Interestingly, TBI has been reported to exacerbate the effects of pesticide exposure, with pesticide‐related PD being substantially higher in individuals who also have a history of TBI [48]. In line with this, Lee et al. [49] reported that while TBI and pesticide exposure in isolation were each associated with only a moderately increased risk of PD development, dual exposure increased the risk three‐fold. This was further corroborated by the results of a rodent study, where a moderate TBI induced by lateral fluid percussion in adult rats resulted in 15% dopaminergic neuron loss ipsilaterally, an effect which was exacerbated by additional exposure to pesticide (30% dopaminergic neuron loss bilaterally) [16]. Thus, pesticide exposure may be a critical environmental risk factor to consider in conjunction with TBI history when investigating PD progression.

Similarly, genetic factors may also interact synergistically with a history of TBI to influence the development or progression of PD. In support of this, a randomised controlled trial reported significant interactions between a history of TBI and polymorphisms in the REP1 gene, with a combination of these factors corresponding to a significantly younger age of disease onset compared to individuals with no such history [50]. Interestingly, however, head injury alone was not significantly associated with PD in that study [50], further highlighting that TBI in isolation may not be sufficient to influence PD progression. The potential utility of assessing synergisms was highlighted by Brown et al. [51], where they found in 378 PwP, occupational pesticide exposure was shown to correspond to a higher risk of balance and cognitive impairments, as well as overall functional impairments in LRRK2‐PD and GBA‐PD respectively. To date, however, literature exploring such synergistic effects between TBI and other known risk factors for PD is scarce, indicating an important avenue for future research.

Present findings should be interpreted with several limitations in mind. Of note, there were significant inconsistencies in the way that TBI characteristics were assessed and obtained across cohorts, such as age at the time of injury. Given that paediatric TBI and adult TBI are considered distinct, which may have critical implications for clinical trajectories, age at the time of injury and the duration of time between injury and assessment should be accounted for as potential predictors of disease progression in PD and other neurodegenerative disorders [52]. However, only a subset of individuals with a history of TBI within the PPMI cohort were able to provide sufficient information on time since injury, with this not considered a variable of interest within the PostCEPT cohort. This is an important oversight and ties directly to another limitation in both datasets, the utilisation of self‐report questionnaires to ascertain history of TBI, which are not considered to be as robust as other gold‐standard practices, including physician‐based diagnosis or standardised interviews. Of note, several standardised assessments designed for retrospective identification and characterisation of TBI exist, each demonstrating high reliability and validity, including the Ohio State University TBI Identification Method [53], Traumatic Brain Injury Questionnaire [54] and Boston Assessment of Traumatic brain Injury Lifetime semi‐structured Interview [55], each of which may have offered a better alternative and should be considered for incorporation in future studies. Finally, it is important to note that the generalisability of findings within the present study may be limited, given the varying definitions of injury severity utilised in the field. For example, while the cut‐off between mTBI and M/sTBI within the present study was LoC of 60 min, other criteria, such as the American Congress of Rehabilitation Medicine Diagnostic Criteria for Mild Traumatic Brain Injury [56] and the Veteran Affairs/Department of Defence guidelines [57] define the cut‐off as 30 min. Taking such limitations into consideration in future studies will allow for more consistent and robust assessment of how TBI and relevant characteristics may influence clinical presentation in PD, as well as other disorders, improving the translatability of research findings across studies.

## 5 Conclusion

While a known risk factor for the later development of PD, present findings do not support that a history of TBI changes the progression of either disease‐related pathology or clinical presentation in those with established PD in two independent cohorts. It is important to acknowledge, however, limitations associated with the current study. Further research aiming to evaluate the effects of TBI itself, as well as several important associated characteristics, such as injury severity, number of injuries, and age at the time of injury, on PD progression should employ a purposely designed study, rather than the convenience samples utilised here, to adequately assess these questions. Further, given the small amount of variance explained in our regression models incorporating only a history of TBI and key demographic variables, it suggests that other factors, or combinations of factors, may contribute to predicting disease progression. Therefore, future work should investigate other known genetic and environmental risk factors for PD, as well as any potential synergistic effects on PD progression.

## Author Contributions

**Angus McNamara:** conceptualization, methodology, formal analysis, writing – original draft, investigation, project administration. **Irina Baetu:** writing – review and editing, methodology, supervision. **Lyndsey Collins‐Praino:** supervision, conceptualization, investigation, writing – review and editing, methodology, project administration.

## Ethics Statement

The PPMI study was approved by the local Institutional Review Boards of respective institutions (a full list is available at the following link https://www.ppmi‐info.org/about‐ppmi/ppmi‐clinical‐sites).

## Consent

Written informed consent was obtained from each participant at enrolment, in accordance with the Declaration of Helsinki. All methods were performed in accordance with the relevant guidelines and regulations. We confirm that we have read the Journal's position on issues involved in ethical publication and affirm that this work is consistent with those guidelines.

## Conflicts of Interest

The authors declare that there are no conflicts of interest relevant to this work. PPMI—a public‐private partnership—is funded by the Michael J. Fox Foundation for Parkinson's Research and funding partners, including 4D Pharma, Abbvie, AcureX, Allergan, Amathus Therapeutics, Aligning Science Across Parkinson's, AskBio, Avid Radiopharmaceuticals, BIAL, Biogen, Biohaven, BioLegend, BlueRock Therapeutics, Bristol‐Myers Squibb, Calico Labs, Celgene, Cerevel Therapeutics, Coave Therapeutics, DaCapo Brainscience, Denali, Edmond J. Safra Foundation, Eli Lilly, Gain Therapeutics, GE HealthCare, Genentech, GSK, Golub Capital, Handl Therapeutics, Insitro, Janssen Neuroscience, Lundbeck, Merck, Meso Scale Discovery, Mission Therapeutics, Neurocrine Biosciences, Pfizer, Piramal, Prevail Therapeutics, Roche, Sanofi, Servier, Sun Pharma Advanced Research Company, Takeda, Teva, UCB, Vanqua Bio, Verily, Voyager Therapeutics, the Weston Family Foundation, and Yumanity Therapeutics. Full financial disclosures for the previous 12 months: AM is supported by an Australian government research training programme scholarship.

## Supporting information


Table S1.

Table S2.

Table S3.

Table S4.

Table S5.

Table S6.

Figure S1.

## Data Availability

The data that support the findings of this study are openly available in Parkinson's Progression Markers Initiative and the Parkinson Research Examination of CEP‐1247 trial (PRECEPT) follow‐up PostCEPT observational study (PostCEPT) at https://www.ppmi‐info.org/access‐data‐specimens/download‐data and https://www.ninds.nih.gov/current‐research/research‐funded‐ninds/clinical‐research/archived‐clinical‐research‐datasets, respectively.
