# Supplementary material for: History of Traumatic Brain Injury Does Not Influence Rate of Progression of Clinical or Pathological Outcomes in Two Early Parkinson's Disease Cohorts
Source: Eur J Neurol. 2025 Mar 20;32(3):e70090. doi: 10.1111/ene.70090 (PMC11926254; doi:10.1111/ene.70090)
Supplement: Supplementary file 1 — Table S1. [file ENE-32-e70090-s004.docx]

| **Inclusion Criteria** | **Exclusion Criteria** |
| --- | --- |
| 1) Presence of 2 or more cardinal motor symptoms:   1. Resting tremor 2. Bradykinesia 3. Rigidity   OR   1. Asymmetric resting tremor   OR   1. Asymmetric bradykinesia   2) Dopamine transporter deficit on SPECT imaging  3) Diagnosis of PD < 2 years before baseline visit | First-degree relative of a PD patient (Genetic cohort)  Scans without evidence of dopaminergic deficit (SWEDD cohort)  < 30 years old |

***Table S1:*** *Inclusion and exclusion criteria PPMI have developed to categorise participants into the early PD cohort.*
