# Supplementary material for: History of Traumatic Brain Injury Does Not Influence Rate of Progression of Clinical or Pathological Outcomes in Two Early Parkinson's Disease Cohorts
Source: Eur J Neurol. 2025 Mar 20;32(3):e70090. doi: 10.1111/ene.70090 (PMC11926254; doi:10.1111/ene.70090)
Supplement: Supplementary file 2 — Table S2. [file ENE-32-e70090-s005.docx]

| PDRFQ Domain | Measures |
| --- | --- |
| Head Injury Questionnaire | 1. **Have you ever had a head injury or concussion?** These may have occurred during sporting activities, from falls, violence, car accidents, or other accidents. Include injuries from both childhood and adulthood  - Yes - Possibly - No (Skip to next form) - Don’t know (Skip to next form) - Refused (Skip to next form)  1. **In your lifetime, how many have you had?**  - 1 - 2 - 3 - 4 - More than 4   *The below questions were repeated depending on the number of head injuries a participant sustained*   1. **At what age (or in what year) did the head injury occur?**  - Age   OR   - Year - Don’t know  1. **Did you lose consciousness from this injury?**  - Yes - Possibly - No (Skip to next form) - Don’t know (Skip to question 6) - Refused (Skip to question 6)  1. **How long were you unconscious?**  - Less than 5 minutes - 5 – 59 minutes - 1 – 24 hours - > 24 hours - Don’t know  1. **Did you have a skull fracture from this injury?**  - Yes - No - Don’t know - Refused  1. **Did you have a seizure from this injury?**  - Yes - No - Don’t know - Refused  1. **Did you have memory loss, amnesia or trouble thinking from this injury?**  - Yes - No - Don’t know - Refused  1. **Were you hospitalised from this injury?**  - Yes - No - Don’t know - Refused |

***Table S2:*** *Head Injury Questionnaire component of the Parkinson’s Disease Risk Factor Questionnaire*
