# Supplementary material for: History of Traumatic Brain Injury Does Not Influence Rate of Progression of Clinical or Pathological Outcomes in Two Early Parkinson's Disease Cohorts
Source: Eur J Neurol. 2025 Mar 20;32(3):e70090. doi: 10.1111/ene.70090 (PMC11926254; doi:10.1111/ene.70090)
Supplement: Supplementary file 3 — Table S3. [file ENE-32-e70090-s002.docx]

| **Inclusion Criteria** | **Exclusion Criteria** |
| --- | --- |
| > 30 years old  Presence of at least two of the cardinal signs of PD (resting tremor, bradykinesia, rigidity)  Modified Hoehn and Yahr stage ≤2.5  No current or imminent (in next 3 months) PD disability requiring dopaminergic therapy | Atypical or drug-induced parkinsonism  Diagnosis of PD ≥5 years duration  Rest tremor score ≥3 in any limb  Use of antiparkinsonian therapy within the past 6 months  History of malignant melanoma  History of cancer (excluding basal and squamous cell skin carcinomas) within the past 5 years  A history of seizures (not including febrile seizures)  Mini-Mental State Examination score ≤26  Beck depression score ≥15  Creatinine clearance ≤50 mL/min  Participation in another investigational drug trial within 60 days of randomisation or in a trial using CEP-1347  Use of medication with potential interactions with CEP-1347  Clinically significant finding on laboratory tests or electrocardiograms  Unstable medical or psychiatric conditions that could adversely affect study participation |

***Table S3:*** *Inclusion and exclusion criteria for the PostCEPT PD cohort.*
