# Supplementary material for: History of Traumatic Brain Injury Does Not Influence Rate of Progression of Clinical or Pathological Outcomes in Two Early Parkinson's Disease Cohorts
Source: Eur J Neurol. 2025 Mar 20;32(3):e70090. doi: 10.1111/ene.70090 (PMC11926254; doi:10.1111/ene.70090)
Supplement: Supplementary file 4 — Table S4. [file ENE-32-e70090-s003.docx]

|  | **Loadings** | **Eigenvalue** | **Variance Accounted by Factor** |
| --- | --- | --- | --- |
| **Baseline Cognitive Ability PCA (PPMI)** |  | 1.97 | 39% |
| *HVLT*  *LNS*  *SFT*  *BJLOT*  *SDM* | .69  .71  .69  .22  .69 |  | |
| **Baseline Mood Dysfunction PCA (PPMI)** |  | 1.65 | 83% |
| *STAI*  *GDS* | .91  .91 |  | |
| **Cognitive Ability Beta Coefficient PCA (PPMI)** |  | 1.70 | 34% |
| *HVLT slope*  *LNS slope*  *SFT slope*  *BJLOT slope*  *SDM slope* | .64  .61  .66  .39  .58 |  | |
| **Mood Dysfunction Beta Coefficient PCA(PPMI)** |  | 1.58 | 79% |
| *STAI slope*  *GDS slope* | .89  .89 |  | |
| **Year-1 Cognitive Ability PCA (PostCEPT)** |  | 1.85 | 62% |
| *HVLT*  *LNS*  *SFT* | .78  .83  .74 |  | |
| **Cognitive Ability Beta Coefficient PCA (PostCEPT)** |  | 1.29 | 43% |
| *HVLT slope*  *LNS slope*  *SFT slope* | .64  .72  .59 |  | |

**Table S4:** *PCA was conducted on cognitive assessments and mood-related assessments to provide composite scores based on the first unrotated factor. This was conducted on baseline values, as well as on beta coefficients that estimated the slope of change in cognitive ability or mood dysfunction over the follow-up period. PCA was not conducted to derive a mood dysfunction composite score in the PostCEPT cohort as only GDS information was available.*
