# Supplementary material for: History of Traumatic Brain Injury Does Not Influence Rate of Progression of Clinical or Pathological Outcomes in Two Early Parkinson's Disease Cohorts
Source: Eur J Neurol. 2025 Mar 20;32(3):e70090. doi: 10.1111/ene.70090 (PMC11926254; doi:10.1111/ene.70090)
Supplement: Supplementary file 5 — Table S5. [file ENE-32-e70090-s001.docx]

|  | **Age at Time of Injury** | | | |  |
| --- | --- | --- | --- | --- | --- |
|  | **Paediatric TBI (≤ 18 years)** | | **Adult TBI (> 18 years)** | |  |
|  | **n** | **Mean ± SD / Median (IQR)** | **n** | **Mean ± SD / Median (IQR)** | **p-value** |
| **Age** | 49 | 59.39 ± 10.99 | 19 | 59.85 ± 10.31 | 0.60 |
| **Education** | 49 | 18 (16-18) | 19 | 16 (15 – 17) | **0.03** |
| **Sex** |  |  |  |  |  |
| Male | 33 (67%) | | 13 (68%) | | 0.99 |
| Female | 16 (33%) | | 6 (32%) | |  |

**Table S5:** *Demographic information of TBI cohort stratified according to whether they sustained a paediatric (≤ 18 years of age at time of injury) or adult TBI (> 18 years of age at time of injury. Significant differences between groups are denoted by bolded p-values*
