# Supplementary material for: History of Traumatic Brain Injury Does Not Influence Rate of Progression of Clinical or Pathological Outcomes in Two Early Parkinson's Disease Cohorts
Source: Eur J Neurol. 2025 Mar 20;32(3):e70090. doi: 10.1111/ene.70090 (PMC11926254; doi:10.1111/ene.70090)
Supplement: Supplementary file 6 — Table S6. [file ENE-32-e70090-s007.docx]

| **PostCEPT Cohort** | | | | | | | | |
| --- | --- | --- | --- | --- | --- | --- | --- | --- |
|  | **Year 4 Cognitive Ability** | | **Year 4 GDS** | | **Year 4 Rigidity Score** | | **Year 4 Tremor Score** | |
| *Predictors* | *Estimates* | *p* | *Estimates* | *p* | *Estimates* | *p* | *Estimates* | *p* |
| Intercept | 1.20 | **0.045** | -0.37 | 0.790 | -1.40 | 0.504 | 1.80 | 0.364 |
| Age | -0.04 | **<0.001** | 0.03 | 0.079 | 0.08 | **0.003** | 0.01 | 0.582 |
| Female Sex | 0.34 | **0.028** | -0.14 | 0.707 | -1.31 | **0.019** | -0.41 | 0.439 |
| Education | 0.18 | **<0.001** | -0.07 | 0.496 | 0.08 | 0.603 | 0.03 | 0.865 |
| History of TBI | -0.12 | 0.485 | -0.38 | 0.357 | 0.33 | 0.590 | 0.43 | 0.460 |
| Baseline GDS Score | -0.02 | 0.580 | 0.73 | **<0.001** | 0.09 | 0.397 | 0.01 | 0.901 |
| Observations | 154 | | 162 | | 162 | | 162 | |
| R^2^ / R^2^ adjusted | 0.267 / 0.242 | | 0.411 / 0.392 | | 0.114 / 0.086 | | 0.013 / -0.019 | |

**Table S6**: *Within the PostCEPT cohort, individuals with a history of TBI demonstrated significantly higher depression scores, measured via GDS. Therefore, exploratory models were conducted to determine whether a history of TBI was a significant predictor of outcome, while controlling for age, sex and baseline GDS scores.*
