# Supplementary material for: History of Traumatic Brain Injury Does Not Influence Rate of Progression of Clinical or Pathological Outcomes in Two Early Parkinson's Disease Cohorts
Source: Eur J Neurol. 2025 Mar 20;32(3):e70090. doi: 10.1111/ene.70090 (PMC11926254; doi:10.1111/ene.70090)
Supplement: Supplementary file 7 — Figure S1. [file ENE-32-e70090-s006.docx]

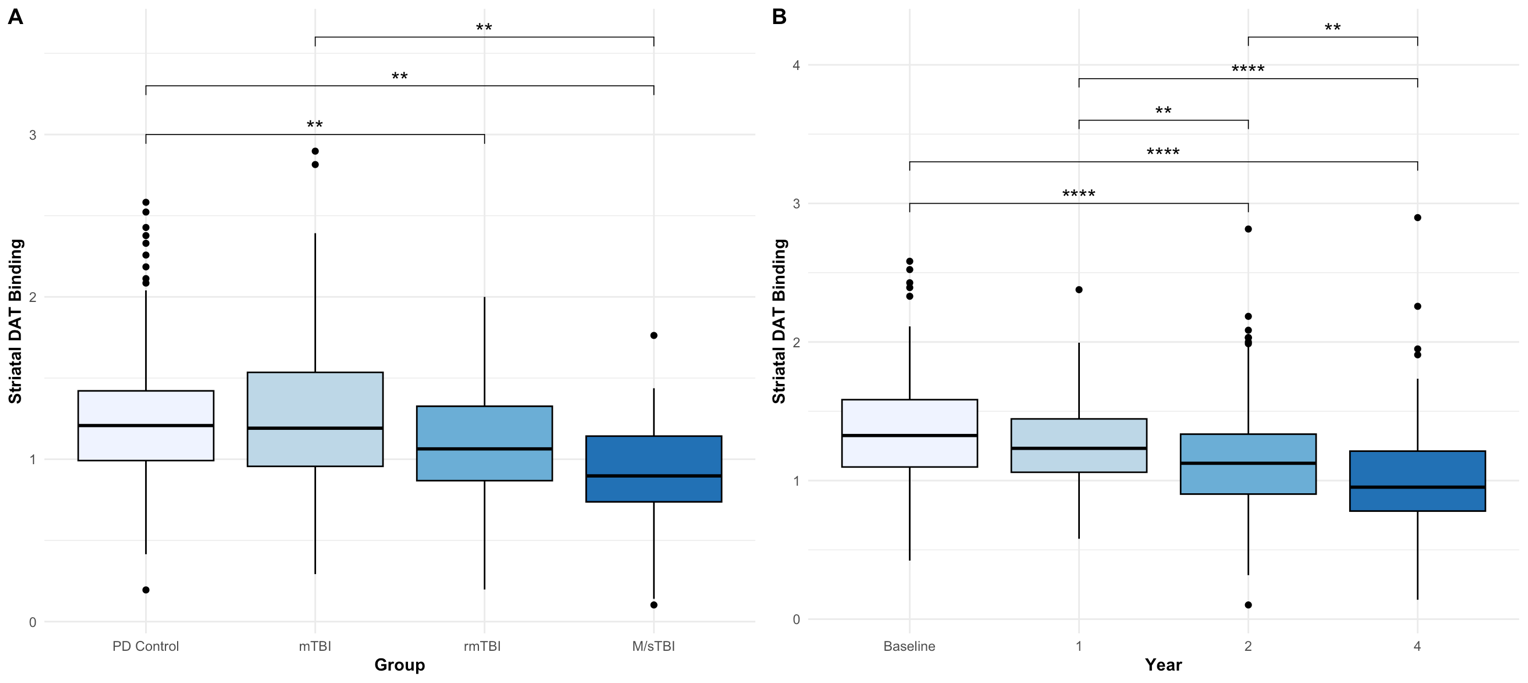


**Figure S1:** *Mixed ANOVA’s determined significant main effects for (1A) TBI severity and 1B) follow-up timepoint for striatal DaT binding. An increase in injury severity and a longer follow-up timepoint were associated with significantly lower striatal DaT binding.*
